# High sugar-sweetened beverage intake frequency is associated with smoking, irregular meal intake and higher serum uric acid in Taiwanese adolescents

**DOI:** 10.1017/jns.2020.2

**Published:** 2020-02-10

**Authors:** Y. H. Shih, H. Y. Chang, H. C. Wu, F. F Stanaway, W. H. Pan

**Affiliations:** 1 Institute of Population Health Sciences, National Health Research Institutes, Miaoli, Taiwan; 2 National Yang-Ming University, Taipei, Taiwan; 3 Department of Food Nutrition, College of Human Science and Technology, Chung Hwa University of Medical Technology, Tainan, Taiwan; 4 Sydney School of Public Health, The University of Sydney, Camperdown, NSW 2006, Australia; 5 Institute of Biomedical Sciences, Academia Sinica, Taipei, Taiwan

**Keywords:** Sugar-sweetened beverages, Food frequency questionnaires, Serum uric acid, Adolescents, Nutrition and Health Survey in Taiwan, HFCS, high-fructose corn syrup, HH, high tea (≥7 times/week) and high soda/sports drink intake (≥4·5 times/week), HL, high tea (≥7 times/week) and low soda/sports drink intake (<4·5 times/week), LH, low tea (<7 times/week) and high soda/sports drink intake (≥4·5 times/week), LL, low tea (<7 times/week) and low soda/sports drink intake (<4·5 times/week), NAHSIT, Nutrition and Health Survey in Taiwan, SSB, sugar-sweetened beverages

## Abstract

Types of sugar-sweetened beverages (SSB) can differ greatly between countries, with greater consumption of sweetened tea in Asia. This study aimed to understand changes in SSB consumption by adolescents in Taiwan over 18 years and their association with demographic characteristics and clinical outcome. This study used survey data from the 1993–1996 and 2010–2011 Nutrition and Health Surveys in Taiwan. Participants were high school students aged 13 to 18 years. Data were weighted and analysed using SUDAAN 11.0 and SAS 9.4. Participants were asked about intake frequencies of SSB and were grouped into four different SSB intake groups based on the combination of high or low frequency (including moderate frequency) of intake of sweetened tea and soda/sports/energy drinks. Results indicated over 99 % of teens reported having at least one SSB in the past week. Smoking status was significantly associated with SSB intake types with high tea intake (high tea and low soda (HL) group, OR 7·56, *P* < 0·001; high tea and high soda (HH) group, OR 9·96, *P* < 0·001). After adjustment for potential confounders, adolescents in the low tea and high soda (LH) group (*β* = 0·05, *P* = 0·034) had significantly higher mean serum uric acid values. In conclusion, sugary tea remains the SSB of choice for Taiwanese adolescents. Those with a frequent intake of soda/sports/energy drinks had a higher chance of being hyperuricaemic.

Overconsumption of sugar-sweetened beverages (SSB) increases the risk of non-communicable diseases including obesity, gout, CVD, fatty liver, insulin resistance and diabetes^(1,2)^. Past research has found that adolescents are more likely to have greater intake of SSB than children and adults^(3–5)^ and that SSB provide about 20–25 % of total sugar intake^(6,7)^. Despite a decline in SSB consumption by adolescents in a number of countries (including Austria, UK and USA)^(8–11)^, consumption remains high. For example, SSB still provided 9·3–9·7 % of total energy intake by US adolescents from 2011 to 2014^(12)^ and 14 % of total energy intake by UK adolescents from 2008^(9)^. The contribution of SSB could be close to or exceeding 10 % which is recommended by the 2015 WHO guidelines^(13)^. The SSB intake rate is very high in Asia; for example, it reached 38 % in Korea^(14)^ and reached 87·7 % in Taiwan in 2009^(15)^. Besides, SSB contributed 25 % of daily intakes of sugar in Japan^(7)^.

An important contributing factor to SSB consumption in Taiwan is its availability. Sweetened beverages are readily obtained from convenience stores as well as beverage shops including cafés, juice stores and tea shops. By 2017, there were an estimated 10 662 convenience stores belonging to the four major chains and 21 346 beverage shops, which equates to an average of one convenience store per 2211 persons and one beverage shop per 1104 persons^(16,17)^. The greater the number of such stores near schools and the shorter walking distance required, the greater the mean daily frequency of SSB purchasing by adolescents^(18)^.

Although a large number of studies have examined SSB intake in adolescents, most are from Western countries and have focused on intake of carbonated drinks (including soda, carbonated soda and carbonated beverages)^(3,19–21)^. Culture plays an important role in the types of beverages consumed by adolescents, with intake of beverages (including coffee and tea) more common in adolescents in Asia and even surpassing that of soda is some Asian countries^(19)^. A survey conducted in 2015 in southern Taiwan showed that SSB contributed 83·5 % of sugar intake, mainly sweetened tea or milk tea (41 %) in teenagers (13–18 years of age)^(22)^. On average, sweetened tea in Taiwan contains 9·19 g/100 ml sugar^(23)^. Normally, servings come in medium (500 ml) or large (700 ml) sizes. If a teenager consumes 9623 kJ/d (2300 kcal/d), about 10 % of the total energy would come from a glass of sweetened tea.

Different types of SSB have different associations with health outcomes. One cross-sectional study showed that excessive intakes of sweetened coffee/tea were associated with larger BMI, waist circumference, and uric acid, whereas the association was not observed in other soft drinks^(24)^. Another study on children aged 6–13 years found that sweetened tea, soft drinks and sports drinks were associated with obesity. There was found to be an association of intakes of sweetened tea with central obesity (OR 1·55; 95 % CI 1·26, 1·90). But this association was not observed in those who drank coffee^(25)^. A study on girls found that drinking soda and sports drinks was positively associated with the amount of fructose intake. On the other hand, drinking coffee/tea was associated with the amount of sucrose intake^(26)^. Besides, beverage shops, which sell hand-made beverages mostly, increased sharply in Taiwan. Therefore, we separated the types of SSB for analyses.

As the above research shows, SSB are an important potential health risk for Taiwanese adolescents. Moreover, it emphasises the importance of separating ‘tea’ as a unique beverage type in this setting. The aim of the present study was to examine changes in SSB consumption by adolescents in Taiwan over 18 years using nationally representative survey data, and to examine associated changes in anthropometric measures and blood biochemistry. Tea beverages were examined as a unique category and participants were grouped into types of SSB consumption based on high or low intakes of sweetened tea and soda/sports/energy drinks.

## Experimental methods

### Participants

The present study analysed secondary data. Two separate cross-sectional datasets for the present study come from the 1993–1996 and 2010–2011 Nutrition and Health Survey in Taiwan (NAHSIT), whose youth data are available for application. The sampling population for the 1993–1996 survey was Taiwanese residents aged 3 years and over with a survey completion rate of 74 %. In the 2010–2011 survey, the sample population was high school students from public and private schools with a completion rate of 87 % in junior high school students (similar to grades 7 to 9; age range 12–15 years old) and 92 % in senior high school students (similar to grades 10 to 12; age range 15–18 years old). Further details about survey participants and sampling method have been described previously^(27,28)^. The present study includes adolescents at junior or senior high school aged 13 to 18 years. Adolescents aged 12 years were excluded because of incomplete data. Data collected included a FFQ, anthropometry and blood samples. Extreme values that were over the lower or upper outer fence in box plots were removed (*n* 186, 4·17 %), which included excessively high (≥41 840 kJ) or low (<836·8 kJ) daily energy intake (*n* 10). Each drink had its extreme value, e.g. coffee >10 times/week or tea >50 times/week. SSB intake frequencies (*n* 64) or outlying blood results (*n* 109) were excluded; a total of 4277 participants were included in the analysis (1993–1996 survey, *n* 1788; 2010–2011 survey, *n* 2489).

The present study was conducted according to the guidelines laid down in the Declaration of Helsinki and all procedures involving human subjects were approved by the National Health Research Institutes Research Ethics Committee (ethics no. EC 0990803-R2, EC1000311-R1, EC1060110-E). Written informed consent was obtained from all subjects in 2010–2011 and verbal informed consent was obtained in 1993–1996 (ethical approval was not required in 1993–1996). Verbal consent was witnessed and formally recorded.

### Measurements

Intake of different types of SSB was assessed using a FFQ^(29)^ which asked participants about their frequency of intake of five types of beverages in the past month (every day, every week, every month) including tea, soda/sports/energy drinks, fruit drinks (including <100 % pure juice drinks such as reconstituted and fermented juice), coffee, and other beverages (yogurt drinks, smoothies, shakes and Slurpee). For ease of analysis, we converted all intake frequencies to a proportional intake per week. The five beverage types did not include 100 % pure fruit juice or alcoholic beverages. The mean weekly intake and prevalence of consumption of each beverage type were compared for the two survey time points. We then categorised participants into four groups based on high or low levels of intake of the two most commonly consumed beverage types of tea and soda/sports/energy drinks. High (≥7 times/week for tea and ≥4·5 times/week for soda/sports/energy drinks) and low or moderate (<7 times/week for tea and <4·5 times/week for soda/sports/energy drinks) intake frequencies were defined using the 3rd quartile frequency in 1993–1996. This approach enabled examination of the combined effects of these two beverage types which we believe leads to more accurate results. The resulting four SSB intake groups were: low intake of tea drinks and low intake of soda/sports/energy drinks (LL group), low intake of tea drinks and high intake of soda/sports/energy drinks (LH group), high intake of tea drinks and low intake of soda/sports/energy drinks (HL group), and high intake of both tea drinks and soda/sports/energy drinks (HH group).

Anthropometric measures and blood biochemistry were collected as part of the NAHSIT survey. Anthropometric measures include height, weight, BMI and waist circumference. BMI was calculated as weight in kg divided by height in m^2^. BMI categories was based on the criteria developed by the Health Promotion Administration, Ministry of Health and Welfare. There were different cut-off points for males and females at different ages. Participants were classified into underweight, normal-weight, overweight, and obese^(30)^. Waist circumference was measured at the midpoint between the top of the pelvis and the bottom of the ribs with the participant standing. Venous blood samples were taken from participants after 8 h of fasting. Blood samples were collected in vacutainer tubes and after being centrifuged were stored on dry ice. Then, they were delivered to a central laboratory and frozen at −70°C on the same day for further analyses. Blood results included HDL-cholesterol, LDL-cholesterol, total cholesterol, TAG, fasting blood glucose and serum uric acid.

Other variables obtained from the NAHSIT questionnaire included age, sex, smoking status (never smoker, ever-smoker – includes ex-smoker, occasional smoker and current smoker), alcohol consumption (never drinker, ever-drinker – mean intake of >0 drinks per month), physical activity and total energy intake. Physical activity was converted into weekly metabolic equivalents (MET) based on the method proposed by Ridley *et al*.^(31)^. Because physical activity and total energy intake were heavily skewed to the right, they were dichotomised into low or high comparing the median of 1993–1996. Physical activity was classified into low (<125·5 kJ/kg per h) or high (≥125·5 kJ/kg per h), and total energy intake was classified into low (<7385 kJ/d) or high (≥7385 kJ/d). FFQ also asked about taking meals regularly. If a person answered to taking three meals daily, this person was considered having a regular dietary habit.

### Statistical analyses

Statistical analyses and adjustment for unequal sampling rates were carried out using SAS version 9.4 (SAS Institute Inc.) and SUDAAN version 11.0. The samples were also weighted to make them representative of the population for the given year. Weekly intakes of beverages were described using mean values and 95 % CI. Percentages were used to describe the prevalence of consumption of each beverage type as well as the distribution of participant characteristics by SSB intake type. Also, *t* tests were used to analyse differences in mean intakes between surveys and *χ*^2^ tests were used to compare differences in prevalence between surveys and differences in the distribution of characteristics by SSB intake group. Multinomial logistic regression was used to examine participant characteristics associated with SSB intake group. Multiple linear regression was used to examine associations between SSB intake group and anthropometric measures and blood results after controlling for survey year, age, sex, metabolic equivalents (MET) and total energy intake. As only LDL-cholesterol, carbohydrate, lipid and SFA had a normal distribution, other measures were all log transformed before being entered into models. After removal of outliers and log transformation, the degree of skewness of residuals in regression models approached zero and kurtosis was less than an absolute value of 2. Two-sided *P* values <0·5 were considered statistically significant.

We compared the differences between the removed (*n* 183) and the rest (*n* 4277) and found no differences in age, sex and weight. We also conducted sensitivity analysis comparing the results with (*n* 4460) and without the outliers (*n* 4277). We obtained similar coefficients, but the variation was larger in the dataset with outliers. For example, the OR were 0·054 (95 % CI 0·00, 0·10) *v.* 0·040 (95 % CI −0·03, 0·11) for log-serum uric acid without and with the outliers, respectively.

## Results

Table 1 shows the distribution of participant characteristics in both surveys after weighting. Compared with those participating in survey 1, more junior high school students responded in survey 2 (*P* = 0·001). In addition, survey 2 participants were less likely to be underweight and more likely to be obese (*P* = 0·001), were more likely to have excessive total energy intakes (*P* < 0·001), and were more likely to not eat lunch regularly (*P* = 0·006).

**Table 1.** Characteristics of participants in both survey years (*n* 4277) (Numbers of participants and weighted percentages)

Characteristic	1993–1993 (*n* 1788)		2010–2011 (*n* 2489)		P*
	*n*	Weighted %	*n*	Weighted %	
Age group (*n* 4277)					0·001
Young (junior high school)	1110	49·5	1444	64·3	
Older (senior high school)	678	50·5	1045	35·7	
Sex (*n* 4277)					0·207
Male	894	53·2	1229	51·7	
Female	894	46·8	1260	48·9	
BMI categories (*n* 3255)†					0·001
Underweight	106	12·5	127	5·3	
Normal weight	758	69·0	1436	65·9	
Overweight	106	11·0	292	13·4	
Obese	77	7·5	353	15·3	
Smoking status (*n* 3254)					0·221
Non-smoker	1193	89·3	1729	92·1	
Ever-smoker‡	139	10·7	193	7·9	
Alcohol drinking status (*n* 3471)					0·662
Non-drinker	1213	80·3	1515	79·9	
Ever-drinker‡	336	19·7	407	20·1	
MET/week (kJ/kg per h) (*n* 3433)					0·079
Low (<125·5)	726	50·1	1058	55·3	
High (≥125·5)	797	49·9	852	44·7	
Total energy (kJ/d) (*n* 4277)					<0·001
Low (<7385)	967	50·1	726	28·8	
High (≥7385)	821	49·9	1763	71·2	
Dietary behaviour					
Eats breakfast (*n* 4249)					0·064
Irregularly (<7 d/week)	556	28·4	862	33·2	
Regularly (=7 d/week)	1205	71·6	1626	66·8	
Eats lunch (*n* 4250)					0·006
Irregularly (<7 d/week)	208	10·8	456	16·7	
Regularly (=7 d/week)	1554	89·2	2032	83·3	
Eats dinner (*n* 4249)					0·505
Irregularly (<7 d/week)	169	10·1	311	11·3	
Regularly (=7 d/week)	1592	89·9	2177	88·7	

MET, metabolic equivalents.

* *P* value based on the *χ*^2^ test.

† BMI was calculated as weight in kg divided by height in metres squared. BMI categories were based on the criteria developed by the Health Promotion Administration, Ministry of Health and Welfare. There were different cut-off points for males and females at different ages. Participants were classified into underweight, normal-weight, overweight, and obese^(30)^.

‡ Ever-smoker: including former smokers and current smokers. Ever-drinker: consumes alcohol >0 times per month.

Table 2 shows the mean frequency and prevalence of intake of each SSB group age standardised to the age distribution in 1993–1996. In the 1993–1996 survey, adolescents had a mean frequency of SSB intake of 12·4 times per week and as many as 99·1 % of adolescents drank at least one SSB per week. The mean SSB intake frequency declined to 10·8 times per week in the 2010–2011 survey (*P* = 0·04); however, 99·5 % of adolescents still reported consuming at least one SSB per week. Both the frequency and prevalence of intake were highest for tea drinks in both surveys, followed by soda/sports/energy drinks and other drinks in 1993–1996, and by other drinks and soda/sports/energy drinks in 2010–2011. Taiwanese adolescents consumed tea drinks an average of four times per week in 1993–1996 and this further increased to five times per week in 2010–2011. In contrast, the intake frequency of both soda/sports/energy drinks and fruit drinks more than halved during the same period (*P* < 0·001). The prevalence of tea consumption also increased from 86·4 % in 1993–1996 to 94·9 % in 2010–2011 (*P* = 0·005), whereas that of soda/sports/energy drinks decreased from 79·9 to 76·3 % (*P* = 0·070) and that of fruit drinks decreased from 60·3 to 51·8 % (*P* = 0·015). Prevalence of drinking coffee and other drinks increased significantly. Prevalence increased from 33·2 to 40·3 % (*P* = 0·027) and from 76·4 to 85·3 % (*P* = 0·032) for coffee and other drinks, respectively.

**Table 2.** Weekly frequency of intake and prevalence of consumption of each type of sugar-sweetened beverage (SSB) in both survey years (*n* 4277)* (Mean values and 95 % confidence intervals; percentages of consumers)

Category	1993–1996 (*n* 1788)			2010–2011 (*n* 2489)			*P* for mean†	*P* for %‡
	Times/week		Consumer (%)	Times/week		Consumer (%)		
	Mean	95 % CI		Mean	95 % CI			
SSB	12·4	11, 13·8	99·1	10·8	10·2, 11·4	99·5	0·040	0·230
Tea	4·3	3·6, 5·0	86·4	5·1	4·7, 5·5	94·9	0·041	0·005
Soda/sports/energy drinks	3·4	3·1, 3·7	79·9	1·7	1·6, 1·9	76·3	<0·001	0·070
Fruit drinks	1·7	1·4, 2·0	60·3	0·8	0·7, 0·9	51·8	<0·001	0·015
Coffee	0·5	0·4, 0·7	33·2	0·6	0·5, 0·7	40·3	0·441	0·027
Other drinks	2·5	2·0, 3·0	76·4	2·5	2·2, 2·9	83·5	0·886	0·032

* Values were age standardised to the age distribution of 1993–1996. Soda/sports/energy drinks included soda, sports drinks and energy drinks; fruit drinks refer to non-100 % juice drinks including reconstituted juice and fermented juice; other drinks included yogurt drinks, smoothies, shakes and Slurpee.

† *P* value based on the *t* test for the comparison of means between 2010–2011 and 1993–1996.

‡ *P* value based on the *χ*^2^ test for the comparison of proportions between 2010–2011 and 1993–1996.

Participants were grouped into four SSB intake groups based on whether they had low and moderate (L group) or high (H group) intake of tea and soda/sports/energy drinks (data not shown). The cut-off point was the 3rd quartile of that drink. It was seven times/week for tea, and 4·5 times/week for soda/sports/energy drinks. The most common intake group in adolescents in both surveys was low in tea and low in soda drink intake (LL) (from 57·8 to 64·8 % of adolescents), followed by high in tea and low in soda drink intake (HL) (from 17·3 to 25·2 %). The prevalence in low in tea and high in soda drink (LH) and high in both groups (HH) decreased significantly over 18 years. Prevalence dropped from 14·4 and 10·4 % in 1993–96 to 5·1 and 4·9 % in 2010–11 (*P* < 0·001) for LH and HH, respectively.

Table 3 shows the results of multinomial logistic regression analyses examining associations between participant characteristics and the four SSB intake groups. There were significantly fewer adolescents with LH intake (OR 0·20; *P* < 0·001) and HH intake (OR 0·33; *P* = 0·019) compared with the LL group in 2010–2011. Being an ever-smoker (includes current smokers) was associated with a greater likelihood of HL intake (OR 7·56; *P* < 0·001) and HH intake (OR 9·96; *P* < 0·001). Having high total energy intake (OR 2·50; *P* = 0·017) was also associated with a greater likelihood of HH intake. Not eating breakfast regularly was associated with a greater likelihood of LH intake (OR 1·73; *P* = 0·002).

**Table 3.** Multinomial logistic regression analysis of chance factors by group of tea and soda/sports/energy drink consumption (*n* 4277)* (Weighted percentages, odds ratios and 95 % confidence intervals)

	LL (reference)		LH				HL				HH			
	Weighted %	OR	Weighted %	OR	95 % CI	*P* *	Weighted %	OR	95 % CI	P*	Weighted %	OR	95 % CI	P*
Survey year														
1st survey	64·7	1·00	85·3	1·00			58·5	1·00			81·5	1·00		
2nd survey	35·3	1·00	14·7	0·20	0·13, 0·33	<0·001	41·5	1·38	0·8, 2·37	0·216	18·5	0·33	0·14, 0·80	0·019
Age group														
Young (junior)	55·4	1·00	58·3	1·00			49·2	1·00			53·6	1·00		
Older (senior)	44·6	1·00	41·7	0·97	0·62, 1·50	0·865	50·8	1·04	0·76, 1·41	0·792	46·4	0·72	0·32, 1·59	0·372
Sex														
Male	47·9	1·00	63·1	1·39	0·98, 1·98	0·064	50·8	0·81	0·54, 1·22	0·283	77·0	1·14	0·80, 1·63	0·428
Female	52·1	1·00	36·9	1·00			49·2	1·00			23·0	1·00		
BMI categories														
Underweight	10·7	1·00	13·0	1·01	0·47, 2·19	0·972	5·7	0·73	0·31, 1·75	0·446	4·0	0·45	0·15, 1·33	0·133
Normal weight	65·7	1·00	69·2	1·00			69·1	1·00			77·6	1·00		
Overweight	12·6	1·00	11·0	0·97	0·26, 3·66	0·960	12·9	0·94	0·60, 1·47	0·767	7·4	0·73	0·29, 1·85	0·462
Obesity	11·1	1·00	6·9	0·86	0·37, 2·00	0·693	12·4	1·06	0·68, 1·66	0·773	11·0	1·10	0·59, 2·07	0·735
Smoking status														
Non-smoker	96·6	1·00	87·7	1·00			80·3	1·00			73·4	1·00		
Ever-smoker†	3·4	1·00	12·3	2·12	0·76, 5·86	0·132	19·7	7·56	4·13, 13·83	<0·001	26·6	9·96	4·85, 20·45	<0·001
Alcohol drinking status														
Non-drinker	84·5	1·00	75·8	1·00			72·8	1·00			74·7	1·00		
Ever-drinker†	15·5	1·00	24·2	1·22	0·34, 4·35	0·734	27·2	1·33	0·76, 2·3	0·281	25·3	1·02	0·46, 2·26	0·953
MET per week														
Low (<30)	55·6	1·00	48·6	1·00			49·9	1·00			33·3	1·00		
High (≥30)	44·4	1·00	51·4	0·95	0·48, 1·86	0·866	50·1	1·14	0·6, 2·17	0·664	66·7	1·82	0·73, 4·53	0·172
Total energy (kJ/d)														
Low (<1765)	46·6	1·00	41·8	1·00			38·9	1·00			30·8	1·00		
High (≥1765)	53·4	1·00	58·2	1·18	0·55, 2·53	0·649	61·1	1·20	0·74, 1·93	0·421	69·2	2·50	1·23, 5·12	0·017
Dietary behaviour														
Eats breakfast														
Irregularly (<7 d/week)	27·3	1·00	30·8	1·73	1·29, 2·32	0·002	36·4	1·42	0·64, 3·16	0·354	32·4	1·15	0·63, 2·11	0·607
Regularly (=7 d/week)	72·7	1·00	69·2	1·00			63·6	1·00			67·6	1·00		
Eats lunch														
Irregularly (<7 d/week)	10·8	1·00	13·4	1·36	0·48, 3·82	0·525	17·5	1·33	0·88, 2·00	0·157	14·5	1·67	0·59, 4·72	0·299
Regularly (=7 d/week)	89·2	1·00	86·6	1·00			82·5	1·00			85·5	1·00		
Eats dinner														
Irregularly (<7 d/week)	9·1	1·00	9·3	0·68	0·23, 2·03	0·450	14·8	1·32	0·82, 2·14	0·224	12·0	1·71	0·56, 5·19	0·305
Regularly (=7 d/week)	90·9	1·00	90·7	1·00			85·2	1·00			88·0	1·00		

LL, low tea (<7 times/week) and low soda/sports drink intake (<4·5 times/week); LH, low tea (<7 times/week) and high soda/sports drink intake (≥4·5 times/week); HL, high tea (≥7 times/week) and low soda/sports drink intake (<4·5 times/week); HH, high tea (≥7 times/week) and high soda/sports drink intake (≥4·5 times/week); MET, metabolic equivalent (in kJ/kg per h).

* OR and *P* values were derived from multinomial logistic regression analyses.

† Ever-smoker: includes former smokers and current smokers. Ever-drinker: consumption of alcohol >0 times per month.

Table 4 shows the mean values of anthropometric measures and blood results and different mean values between the two surveys by SBB intake group. All values in Table 4 were adjusted by age prevalence in 1993–1996. Total cholesterol was significantly lower in the HH group compared with the HL group in 1993–1996 (*P* < 0·05), and fasting blood glucose was significantly higher in the LH group compared with the LL group in 2010–2011 (*P* < 0·05). Serum uric acid was highest in the LH group, followed by the HH group in both surveys, and the lowest value was in the LL group. All the comparisons showed that uric acid in LH or HH was higher than that in LL (*P* < 0·05), and it was higher in LH than in HL (*P* < 0·05). In addition, uric acid level in HH was higher than that in HL in 2010–2011 (*P* < 0·05). Statistically significant increases in BMI, waist circumference and fasting blood glucose (*P* < 0·001) were observed between the two surveys. Serum uric acid significantly decreased (*P* < 0·001) compared with the first survey. BMI, waist circumference and fasting blood glucose were significantly different in the four different drinking groups (*P* < 0·05).

**Table 4.** Distribution of anthropometric measures and blood biochemistry by group of tea and soda/sports/energy drink consumption in adolescents (*n* 4277)* (Mean values or mean differences with their standard errors)

Variable	All			LL			LH			HL			HH		
	Mean	se	*P*	Mean	se	*P*	Mean	se	*P*	Mean	se	*P*	Mean	se	*P*
1993–1996 (1st survey)															
BMI (kg/m^2^)	20·2	0·1		20·2	0·3		19·6	0·3		20·4	0·3		20·3	0·4	
WC (cm)	65·6	0·3		65·8	0·6		64·9	1·1		65·3	0·7		66·3	1·5	
HDL-C (mg/dl)ǁ	57·6	1·6		57·6	1·1		55·4	4·6		60·1	2·3		56·6	2·2	
LDL-C (mg/dl)ǁ	90·1	1·7		89·1	2·5		94·9	2·8		91·5	3·1		85·2	4·6	
TC (mg/dl)ǁ	161·5	2·1		160·6	2·3		164·1	3·1		165·3	3·6		154·7	5·3	§
TAG (mg/dl)ǁ	68·3	2·0		68·8	2·6		68·8	3·3		67·9	2·0		63·4	4·2	
FBG (mg/dl)ǁ	80·0	0·8		80·0	0·8		79·9	0·7		79·7	1·3		80·5	0·7	
SUA (mg/dl)ǁ	6·5	0·1		6·3	0·1		7·0	0·2	†	6·4	0·1	‡	6·8	0·2	†
2010–2011 (2nd survey)															
BMI (kg/m^2^)	21·6	0·1		21·5	0·1		22·2	0·5		21·7	0·2		21·8	0·5	
WC (cm)	76·7	0·3		76·2	0·4		78·1	1·3		77·4	0·5		77·4	1·1	
HDL-C (mg/dl)	55·2	0·4		55·7	0·5		52·8	1·5		54·7	0·5		52·5	1·8	
LDL-C (mg/dl)	90·3	0·8		89·8	1·0		96·1	3·1		90·1	1·4		93·2	2·5	
TC (mg/dl)	159·7	0·9		159·6	1·2		164·4	2·7		159·1	1·4		160·8	2·2	
TAG (mg/dl)	71·3	1·1		70·3	1·1		78·3	3·9		72·4	1·7		74·1	3·6	
FBG (mg/dl)	94·6	0·4		94·3	0·4		97·4	1·4	†	94·8	0·6		94·5	1·1	
SUA (mg/dl)	5·8	0·0		5·7	0·0		6·6	0·2	†	5·9	0·1	†‡	6·3	0·1	†§
Mean difference (2nd survey–1st survey)															
BMI (kg/m^2^)	1·4	0·2	<0·001	1·2	0·3	0·003	2·6	0·5	0·001	1·2	0·4	0·008	1·5	0·6	0·041
WC (cm)	11	0·5	<0·001	10·4	0·7	<0·001	13·2	1·7	<0·001	12·1	0·8	<0·001	11·1	1·9	<0·001
HDL-C (mg/dl)	−2·5	1·7	0·164	−1·9	1·2	0·151	−2·6	4·8	0·605	−5·4	2·4	0·045	−4·0	2·9	0·192
LDL-C (mg/dl)	0·3	1·9	0·892	0·7	2·7	0·788	1·3	4·2	0·769	−1·4	3·4	0·685	8·0	5·2	0·158
TC (mg/dl)	−1·0	2·3	0·452	−1·0	2·5	0·711	0·3	4·1	0·938	−6·2	3·8	0·140	6·1	5·7	0·311
TAG (mg/dl)	3·1	2·3	0·216	1·4	2·8	0·624	9·5	5·1	0·095	4·6	2·6	0·114	10·7	5·5	0·082
FBG (mg/d)	14·6	0·9	<0·001	14·3	0·9	<0·001	17·4	1·5	<0·001	15·1	1·4	<0·001	14·0	1·3	<0·001
SUA (mg/dl)	−0·6	0·1	<0·001	−0·6	0·1	<0·001	−0·4	0·3	0·246	−0·5	0·2	0·012	−0·5	0·2	0·086

LL, low tea (<7 times/week) and low soda/sports drink intake (<4·5 times/week); LH, low tea (<7 times/week) and high soda/sports drink intake (≥4·5 times/week); HL, high tea (≥7 times/week) and low soda/sports drink intake (<4·5 times/week); HH, high tea (≥7 times/week) and high soda/sports drink intake (≥4·5 times/week); WC, waist circumference; HDL-C, HDL-cholesterol; LDL-C, LDL-cholesterol; TC, total cholesterol; FBG, fasting blood glucose; SUA, serum uric acid.

* Values have been age standardised to the age distribution of 1993–1996.

† Significantly different from participants in the LL group in the same survey (*P* < 0·05; *t* test).

‡ Significantly different from participants in the LH group in the same survey (*P* < 0·05; *t* test).

§ Significantly different from participants in the HL group in the same survey (*P* < 0·05; *t* test).

ǁ To convert cholesterol from mg/dl to mmol/l, multiply by 0·0259. To convert TAG from mg/dl to mmol/l, multiply by 0·0113. To convert FBG from mg/dl to mmol/l, multiply by 0·0555. To convert SUA from mg/dl to μmol/l, multiply by 59·48.

Table 5 shows the associations between the four SSB intake groups and anthropometric measures and blood biochemistry results. After adjustment for potential confounders, compared with the LL group, adolescents in the LH group (*β* = 0·05; *P* = 0·032) had significantly higher mean serum uric acid values. No other significant associations were observed between SSB intake group and anthropometric measures or blood results. We also examined serum uric acid values stratifying by sex (data not shown) and found that serum uric acid levels were significantly lower in both males and females (*P* < 0·001) in the second survey. Mean serum uric acid decreased from 7·4 to 6·7 mg/dl (440·2 to 398·5 μmol/l) in males, and from 5·5 to 4·8 mg/dl (327·1 to 285·5 μmol/l in females. In both surveys, mean serum uric acid was significantly higher in males compared with females in all of the four SSB intake groups (*P* < 0·05). In the adjusted analysis, no significant differences in serum uric acid between SSB intake groups were observed in males and females.

**Table 5.** Multivariate linear regression analysis of associations of groups of tea and soda/sports/energy drink consumption in adolescents with anthropometric measures and blood biochemistry*† (*β*-Coefficients and 95 % confidence intervals)

Variable‡	LL (reference)	LH			HL			HH		
	*β*	*β*	95 % CI	*P*	*β*	95 % CI	*P*	*β*	95 % CI	*P*
Log-BMI (kg/m^2^)	1·00	−0·004	−0·02, 0·01	0·569	−0·001	−0·01, 0·01	0·863	0·001	−0·02, 0·03	0·912
Log-WC (cm)	1·00	−0·011	−0·05, 0·03	0·597	0·001	−0·01, 0·01	0·806	0·008	−0·02, 0·04	0·552
Log-HDL (mg/dl)	1·00	−0·047	−0·17, 0·08	0·420	0·022	−0·02, 0·07	0·309	−0·012	−0·10, 0·07	0·753
LDL (mg/dl)	1·00	7·337	−1·22, 15·89	0·085	1·951	−3·26, 7·16	0·423	1·780	−3·88, 7·44	0·500
Log-TC (mg/dl)	1·00	0·030	−0·01, 0·07	0·098	0·017	−0·01, 0·04	0·194	0·015	−0·03, 0·06	0·479
Log-TAG (mg/dl)	1·00	0·035	−0·03, 0·10	0·257	0·011	−0·04, 0·06	0·602	0·024	−0·11, 0·15	0·683
Log-FBG (mg/dl)	1·00	0·005	−0·01, 0·01	0·318	0·002	−0·01, 0·02	0·766	−0·003	−0·03, 0·02	0·796
Log-SUA (mg/dl)	1·00	0·054	0·00, 0·10	0·034	0·022	−0·01, 0·06	0·181	0·048	−0·02, 0·11	0·133

LL, low tea (<7 times/week) and low soda/sports drink intake (<4·5 times/week); LH, low tea (<7 times/week) and high soda/sports drink intake (≥4·5 times/week); HL, high tea (≥7 times/week) and low soda/sports drink intake (<4·5 times/week); HH, high tea (≥7 times/week) and high soda/sports drink intake (≥4·5 times/week); WC, waist circumference; TC, total cholesterol; FBG, fasting blood glucose; SUA, serum uric acid.

* *β*-Coefficients are linear regression coefficients derived from multivariable linear regression analyses. All models were adjusted for survey year, age, sex, metabolic equivalent per week (in kJ/kg per h) and total energy intake (in kJ).

† This Table presents results without adjusting for total energy intake. The adjusted analyses were carried out and resulted in similar coefficients.

‡ Apart from LDL, other variables were all log transformed due to lack of a normal distribution.

## Discussion

We observed a decline in frequency of intake of SSB in adolescents over a 14-year period. However, the prevalence of SSB consumption remains high with over 99 % of adolescents consuming at least one type of SSB on a weekly basis. In addition, the prevalence of sweetened tea intake significantly increased and the mean frequency of intake of sweetened tea drinks was as high as five times per week. These findings confirm the importance of measuring tea as a separate beverage type in our population. Frequent consumption of sweetened tea drinks with occasional consumption of soda/sports/energy drinks was the most common intake type (43 % of adolescents in the 2010–2011 survey) and also demonstrated the largest increase in prevalence over time. Smoking status was the chance factor most strongly associated with high SSB intake. In adjusted analyses of anthropometric measures and blood biochemistry, serum uric acid levels were higher in adolescents who frequently consumed soda/sports drinks (regardless of high or low tea drink consumption).

Despite the decline in mean frequency, the prevalence of SSB consumption in our study was high. The prevalence of daily SSB consumption in children and adolescents decreased from 79·7 % in 2003–2004 to 60·7 % in 2014 in the USA^(32)^, and from 76·8 % in 2003 to 71·4 % in 2008 in Australia^(8)^. These trends in SSB intake are probably related to declines in soda drink consumption, energy intake from soda drinks, and the proportion of total energy intake from soda drinks since the year 2000. In contrast, consumption of other beverages such as coffee and tea or sports and energy drinks has increased^(8,10,11)^. In our study, we found that the prevalence of weekly tea intake significantly increased from 94·5 to 99·1 % whereas that of soda/sports/energy drinks decreased from 79·9 to 76·3 %. Similar results have been found in Korea, where the prevalence of drinking soda drinks has declined from 22 % in 2001 to 18 % in 2009 in adolescents. However, the prevalence of overall SSB intake has increased due to increased intake of other beverages (including sports drinks/energy drinks, coffee/tea, flavoured milk and other drinks)^(14)^. A similar phenomenon has been observed in Mexico, where despite the prevalence of SSB consumption increasing from 91·5 % in 1999 to 94 % in 2012, there has been no statistically significant change in the prevalence of soda drink consumption. In contrast, the prevalence of sugar-sweetened coffee or tea intake has significantly increased from 14·2 to 20·8 %^(33)^.

Smoking consumption in adolescents can be associated with intake of SSB^(34,35)^. In our study, smoking status was the only participant characteristic significantly associated with two different SSB intake groups. Both of the intake groups associated with smoking (HL and HH) involved frequent consumption of tea drinks, indicating that those who frequently drink tea beverages are also more likely to smoke. If frequent consumption of soda/sports drinks is added to frequent tea drink consumption, then the likelihood of smoking further increases substantially. Not eating breakfast regularly has been found to be associated with SSB intake^(36–38)^. This association was only found in the low frequent intake of tea and high frequent intake of soda/sports/energy drinks (LH) group in the present study. The association was not significant in other tea drinking groups, HL and HH. It is possible that breakfast sold in the stores always came with tea. Nowadays, many adolescents buy breakfast from the store instead of eating at home^(39–41)^.

In adjusted analyses of anthropometric measures and blood biochemistry, we found that adolescents with low frequent intake of tea and high frequent intake of soda/sports/energy drinks (LH group) had significantly higher serum uric acid levels (*P* < 0·05). Other groups showed positive association with uric acid, but the difference did not reach statistical significance. This difference could be due to the types of sugar added to these beverages. In Taiwan, tea drinks are normally sweetened by adding sucrose (a disaccharide consisting of 50 % fructose and 50 % glucose) or high-fructose corn syrup (HFCS: a monosaccharide consisting of 55 % fructose and 45 % glucose). However, soda, sports drinks and energy drinks are sweetened mainly by adding HFCS. Although both of these sugars are formed from fructose and glucose, HFCS contains more fructose than glucose. Fructose does not have a negative feedback system via ATP and citric acid, which are involved in glucose metabolism. When fructose is stimulated by fructokinase (also known as ketohexokinase), ATP is converted to uric acid, causing a rise in its concentration^(42)^. A previous study also showed that a higher intake of soda was associated with a higher level of uric acid in adults (aged above 19 years)^(43)^. Some studies showed that tea could reduce uric acid levels^(44)^. This was consistent with our finding that adolescents who drank soda more often compared with those who drank tea had high uric acid levels. On the other hand, some studies could not establish a relationship^(44,45)^. However, these studies did not indicate whether sugar was added in tea. Thus, various results have been observed.

Serum uric acid levels in adolescents declined between surveys. However, mean serum uric acid in the group with the most infrequent SSB intake was 5·7 mg/dl (339·0 μmol/l), which is still higher than that observed in other Asian countries such as China (5·27 mg/dl; 313·46 μmol/l)^(46)^ and other Western countries such as the USA, Australia and Italy (4·59–5·52 mg/dl; 273·01–328·33 μmol/l)^(47–49)^. Serum uric acid is an independent chance factor for all-cause mortality^(50,51)^ and is a chance factor for childhood and adolescent chronic kidney disease^(52)^. Taiwan has the highest prevalence and incidence of end-stage renal disease in the world^(53)^. Moreover, from 2005 to 2012 the incidence of end-stage renal disease showed the largest increase in those aged 0 to 19 years^(54)^. Although few studies have linked SSB to end-stage renal disease^(55,56)^, some studies did find an association between SSB and the chance of chronic kidney disease^(57,58)^. As a result, control of uric acid levels and consumption of SSB are of primary importance to adolescent health in Taiwan.

The present study found that uric acid level was associated with SSB intake frequencies only after controlling for other variables. However, other studies have found that SSB intakes were associated with waist circumference^(15,59)^, BMI^(15,59)^, blood pressures^(48,60)^ and TAG^(61)^. This was possible due to culture difference, types of SSB, and ways of estimating SSB amount. This study only analysed the frequency of SSB intakes, and grouped SSB into two major types. That might cause the different results. Studies have shown that drinking tea could lower all-cause mortality, risks of stroke and diabetes, and improve blood pressure, total cholesterol and LDL^(62,63)^. Those studies were on tea without sugar. However, our study was mostly on tea drinks sold in beverage shops, which add sugar. That could cause the different results.

The strengths of the present study include the use of a large-scale dataset that was nationally representative. SSB intake was estimated using a FFQ, which facilitated understanding of the dietary habits of each participant. In addition, we compared measures taken more than 18 years apart which enabled us to better capture behavioural changes over a long time period. The present study is also the first to combine the intake frequency of two different kinds of beverages to investigate SSB intake groups. Our study also has several limitations. (1) As this is a cross-sectional analysis, we are not able to establish causal relationships. (2) As we categorised soda, sports drinks and energy drinks as one beverage type, it was not possible to examine the characteristics of those consuming only one of these types of drinks or to examine comparative changes in the consumption of these different beverages between surveys. However, we were limited by the grouping together of these three beverage types in the first NAHSIT and therefore needed to maintain this grouping when comparing results between surveys. (3) As we used FFQ, we could not estimate intake amounts. The amount of intakes can be obtained through 24-h recall. Most of the time, 1 d measurements were taken. The purpose of this study was to examine SSB intake types. Thus, 24-h recall data were not used. (4) In 1993–1996, the FFQ on SSB did not ask whether it was sweetened or not. At that time, shops did not label the amount of sugar added in the drink. We assumed that most adolescents selected sugar-sweetened drinks.

In conclusion, sugar-sweetened tea was the principal SSB consumed by Taiwanese adolescents. Not only was there a statistically significant increase in the prevalence of consumption of this beverage, but the mean frequency of intake increased from 4·3 times in 1993–1996 to 5 times per week in 2010–2011. High SSB intake was associated with serum uric acid and despite the decline in serum uric acid observed, the level remains high compared with other countries. Therefore, controlling SSB intake in Taiwanese adolescents remains an important public health challenge.
